# Differential Effects of Short-Term Treatment with Two AT_1_ Receptor Blockers on Diameter of Pial Arterioles in SHR

**DOI:** 10.1371/journal.pone.0042469

**Published:** 2012-09-05

**Authors:** Sébastien Foulquier, François Dupuis, Caroline Perrin-Sarrado, Katy Maguin Gatè, Pierre Leroy, Patrick Liminana, Jeffrey Atkinson, Christine Capdeville-Atkinson, Isabelle Lartaud

**Affiliations:** EA3452 CITHEFOR “Drug targets, formulation and preclinical assessment”, Faculté de Pharmacie, Université de Lorraine, Nancy, France; University of Otago, New Zealand

## Abstract

Chronic treatment with angiotensin receptor blockers is largely accepted for protecting cerebral circulation during hypertension, but beneficial effects of short-term treatments are questionable, as highlighted by the recent SCAST trial. We compared the impact of 10 days treatment with candesartan (as SCAST) *versus* telmisartan (previously described to reverse arteriolar remodeling, chronic treatment) on pial arterioles of spontaneously hypertensive rats (SHR). We explored whether PPAR-gamma agonist activity or AT_1_ receptor blockade are involved in their differential effects. In the first study, 4-month-old male SHR were treated with telmisartan (TELMI, 2 mg/kg per day) or candesartan cilexetil (CANDE, 10 mg/kg per day) and compared to vehicle treated SHR and normotensive WKY. In a second study, SHR were treated with CANDE, pioglitazone (a PPAR-gamma agonist, PIO 2.5 mg/kg per day) or CANDE+PIO, compared to TELMI. Internal diameter of pial arterioles (ID, cranial window) was measured at baseline, during hemorrhage-induced hypotension, or following suffusion of Ang II (10^−6^ mol/L) or EDTA inactivation of smooth muscle cells (passive ID). PPAR-gamma and eNOS (target gene of PPAR-gamma) mRNA were evaluated in brain microvessels. For similar antihypertensive effects, TELMI (+44% *versus* SHR), but not CANDE, increased baseline ID. During hemorrhage, ID in TELMI group was similar to WKY, while ID in SHR and CANDE remained lower. In the second study, TELMI (+36%, *versus* SHR) and CANDE+PIO (+43%) increased baseline ID, but not CANDE or PIO alone. TELMI (−66%) and CANDE+PIO (−69%), but neither CANDE nor PIO alone, decreased Ang II-induced vasoconstriction. CANDE+PIO, but not CANDE, increased passive ID. In both studies, PPAR-gamma and eNOS expressions were higher in TELMI than CANDE.

Short-term treatment with TELMI, but not with CANDE, reverses narrowing of pial arteriolar ID in SHR. This may involve PPAR-gamma related mechanisms, since CANDE+PIO treatment induced similar effects, and a better blockade of AT_1_ receptors.

## Introduction

Clinical studies show that chronic treatment with angiotensin II (Ang II) receptor blockers (ARBs) affords protection against cerebrovascular complications [1], [2]. Protective actions of chronic treatment with ARBs on cerebral circulation have been extensively studied in preclinical models. Chronic Ang II blockade reverses hypertension-induced pial arteriolar remodeling in spontaneously hypertensive rats (SHR, a rat model of human hypertension) and exerts strong anti-inflammatory actions [3]–[5].

A chronic treatment with ARBs is largely accepted for protecting cerebral circulation during hypertension and preventing stroke, although beneficial effects of short-term treatments remain questionable as highlighted by the conclusion of the recent SCAST trial [6]. In this trial, 7 days with candesartan cilexetil (CANDE) did not improve post-stroke outcome of hypertensive patients. Thus, short-term treatment with CANDE during the brief post-stroke period does not appear to be beneficial [6]. This highlights a discrepancy between the lack of effect of ARBs after short-term treatment and the beneficial chronic effects. The present study was thus conducted to clarify the short-term impact of ARBs.

On the basis of the above, we compare in SHR, short-term treatments with CANDE (as in SCAST trial [6]) *versus* telmisartan (TELMI), an ARB with previously demonstrated chronic cerebroprotective effects [4]. To our knowledge, comparisons between short-term treatments with ARBs on cerebral arteries are scarce in preclinical studies. We established a 10-day treatment with ARBs on the basis of SCAST trial (7-day treatment with CANDE) and the first observed functional remodeling in cerebral circulation appearing in SHR between 7 to 14 days of treatment [7]. The doses used for CANDE and TELMI were chosen to produce similar anti-hypertensive effects.

In a first study, we compared their impacts on baseline internal diameter (ID) of pial arterioles in SHR (cranial window) and changes of pial arteriolar ID during a hemorrhage-induced fall in arterial pressure. This highlighted a beneficial effect of TELMI but not of CANDE. As TELMI exerts a PPAR-gamma agonist activity [8], [9], we evaluated PPAR-gamma and eNOS expressions (eNOS is a target gene for PPARgamma activity [10], [11]) in brain microvessels. With TELMI, PPAR-gamma expression was maintained at the level of SHR and the eNOS tended to increase. This was not the case with CANDE.

We thus conducted a new set of experiments in order to elucidate the mechanisms involved in the short-term beneficial cerebrovascular effect of TELMI. One explanation could be related to the PPAR-gamma agonist activity. Such activity has been described for TELMI at plasma concentrations achieved with similar conventional antihypertensive dosing as in the present studies [8]. PPAR-gamma activity is responsible for structural and functional remodeling of extra-cerebral vessels [12], [13] and could exert beneficial actions at the cerebrovascular level together with anti-oxidative properties [14], [15]. Such PPAR-gamma activity cannot be induced by CANDE [8]. We thus initiated a strategy based on supplementation of CANDE with the PPAR-gamma agonist pioglitazone (PIO). We analyzed the effects of CANDE and PIO alone or in combination on structure and vasoreactivity of pial arterioles of SHR, and on glutathione content (marker of oxidative status) of brain microvessels of SHR. TELMI was used as a positive control.

A second explanation could be related to a difference in AT_1_ receptor blockade. As this may participate to the different effects of treatments, we studied vasoreactivity of pial arterioles to Ang II. We analyzed levels of angiotensin receptors expression together with plasmatic concentrations of ARBs.

## Methods

### Ethics Statement

All experiments were performed in accordance with the European Community guidelines (2010/63/EU) for the use of experimental animals, the respect of the 3 Rs' requirements for Animal Welfare (I. Lartaud permit number n° 54-5, F. Dupuis n° 54–105, French Ministry of Agriculture, Paris, France). The protocols and procedures applied were similar to those approved by an advisory Regional Ethical Committee on Animal Experiments (CREEA Nancy – Lorraine – Nord-Est, Dr. J. Barrat, June 2, 2008) for another study [16].

### Animals and treatments

The experiments were conducted on 4 to 5-month-old male SHR and normotensive Wistar-Kyoto rats (WKY, Janvier, Le Genest-Saint-Isles, France). Rats of the first study were treated for 10 days by daily gavage with CANDE (10 mg/kg per day, 10 mL/kg, in 5% arabic gum suspension) or TELMI (2 mg/kg per day) dissolved in drinking water containing mannitol 8.10^−4^ mol/L+NaOH 6.10^−5^ mol/L [4].

In the second study, rats received CANDE, or PIO (2.5 mg/kg per day, 5 mL/kg, in 0.5% carboxymethyl cellulose solution) [12] alone or in combination (CANDE+PIO, 10 mg/kg per day +2.5 mg/kg per day). Another group receiving TELMI served as positive control.

Fluid intake and body weight were recorded twice a week in order to adapt concentrations of TELMI and its solvents. Control SHR, WKY and TELMI were treated by gavage with water (10 mL/kg) and all the groups received drinking water with solvents for TELMI.

The doses of ARBs were chosen to induce an equal reduction in systemic mean arterial pressure [4], [17], the dose of PIO on the basis of a protective effect on the aortic wall with no change in arterial pressure [12]. The present doses of PIO and TELMI were previously shown to activate PPAR-gamma *in vivo* [18], [19].

### Maintenance of anesthesia (cranial window experiment)

Anesthesia was maintained by continuous infusion of sodium pentobarbitone (20 mg/kg per hour). The local ethic committee evaluated that pain was low (grade 1 of the classification of the Swiss Federal Veterinary Office) in such experimental protocols and that the use of pentobarbitone was adequate for the management of anesthesia. We assessed the adequacy of anesthesia every 15 minutes by (i) loss of the corneal reflex when touching the cornea, (ii) loss of the withdrawal reflex of the limb when pinching the toe, indicating whether the animal feels pain or not, and (iii) continuous monitoring of systemic arterial pressure (blood pressure should be stable even when testing the withdrawal reflex). If a change in blood pressure or one of the reflexes occurred, a bolus of pentobarbitone was immediately administered intravenously (30 mg/kg) and the flow of infusion adapted (20 to 30 mg/kg per hour).

### Hemodynamics and measurement of reactivity of pial arterioles

Systemic mean arterial blood pressure (femoral artery, MAP, mmHg) and heart rate (bpm) were measured in anesthetized (sodium pentobarbitone, see above) and artificially ventilated animals (for initial values of pH and arterial blood gases, please see online supplement, Table S1). ID of pial arterioles (6–7th order from middle cerebral artery) was measured using an open cranial window technique [4], [16], [20], [21]. Baseline systemic MAP (mmHg), heart rate (bpm), ID (µm), pH and blood gases were measured thirty minutes after completion of the 2-hour surgery. pH and blood gases did not change during the 3-hour experiment (data not shown).

In the first study, ID changes in response to low input blood pressure were evaluated using stepwise hypotension induced by withdrawal of blood from baseline systemic MAP in 10 mmHg steps down to 30 mmHg. Cerebral arteriolar ID increased at each stepwise fall in pressure, consistent with the autoregulation properties [22]. After the final step, rats were sacrificed (sodium pentobarbitone, 250 mg/kg, i.v.).

In the second study, responses to Ang II (10^−6^ mol/L) were evaluated as changes in ID measured after 15-min peri-arteriolar application [16], [20]. Responses to serotonin (5-HT, 10^−6^ mol/L) and adenosine diphosphate (ADP, 10^−4^ mol/L) in presence of serotonin were evaluated after 5-min peri-arteriolar application. Drugs were applied directly in the cranial window at sub-maximal concentrations [16], [20], [23]. The cranial window was washed with artificial cerebrospinal fluid for 15 min between each substance, allowing ID to return to baseline values (results not shown). We used Ang II at 10^−6^ mol/L as it induces 80% of maximal effect as previously shown on a complete concentration response curve (CRC) in normotensive Wistar rats [20]. We did not check whether CRC may differ in SHR, however, as the objective of this protocol was to evaluate changes in response when the arterioles come from rats previously treated with ARBs, complete CRC would not give further information.

Both studies were performed in a simple-blind approach. The researcher was blind to the group when performing the cranial window.

### Mechanics and structure of pial arterioles

Mechanics and structure of pial arterioles were investigated (second study) following inactivation of vascular smooth muscle cells (EDTA 67 mmol/L) [4], [16], [21]. Pial arteriolar blood pressure (PiAP) was continuously measured with a servo-null pressure-measuring device (model 5A, Vista Electronics Company, Ramona, California). Arteriolar pressure-diameter relationships were evaluated during a hemorrhage-induced hypotension. Blood was reinfused after the last arteriolar pressure step and arterioles were fixed with glutaraldehyde (2.25% v/v in 0.1 mol/L cacodylate buffer). After sacrifice (sodium pentobarbitone, 250 mg/kg), the arteriolar segment was set in resin and cross sectional area (CSA, µm^2^) measured in 1 µm thin sections. Wall thickness (WT) and the slope of the stress-strain curve *i.e.* slope of the tangential elastic modulus (ET), which reflects distensibility of the arteriolar wall, were calculated for each pressure step using values of ID obtained during hemorrhage and CSA [4], [16], [21].

### Isolation of brain microvessels

In both studies, brain microvessels were isolated from anesthetized rats (sodium pentobarbitone, 60 mg/kg, i.p.), in which brain was perfused with saline solution. Rats were then sacrificed (sodium pentobarbitone, 250 mg/kg, i.v.), their brain removed, rinsed and homogenized in isotonic sucrose buffer at 4°C [24]. Homogenate was submitted to successive homogenisation/centrifugation cycles. The degree of purification of the microvessels was checked by microscope and gamma-glutamyl transpeptidase (GGT) activity, a marker enzyme mainly localized to endothelial cells of brain microvessels. According to Yamakawa *et al*., [25], the GGT activity should be at least 10 times higher in brain microvessels than in whole brain homogenates for highly purified brain microvessels. Microscopic observation revealed tubular structures, representing well preserved vascular complexes with diameters ranging from that of capillaries to small arterioles. The microvessel preparations were free of contamination by neuronal cells or other cell fragments and, as expected, their GGT activity was higher than in whole brain homogenates (WKY 11±1, SHR 10±1, p>0.05).

### Measurement of reduced glutathione

Glutathione plays a major antioxidant role in the brain [24], [26] and its decrease constitutes a marker of oxidative stress. We previously described an assay based on the use of 2,3-naphthalenedicarboxyaldehyde (NDA), a GSH-selective fluorogenic probe, and inhibition of GGT [24], allowing to measure reduced glutathione in very small samples rich in GGT activity such as brain microvessels. Each aliquot of sample (n = 5 rats per group) or GSH standard was measured in triplicate. The fluorescence intensity of GSH-NDA adduct was measured at λ = 528 nm [24]. The GSH content in samples was calculated from the equation of the calibration curve, and expressed as nmol GSH per mg of protein (protein content determination by the Lowry method).

### Measurement of AT_1_ and AT_2_ receptor protein levels

Microvessels were lysed for 20 min at 4°C in 3 mM HEPES, pH 7.4, containing 1% sodium dodecyl sulfate, protease inhibitor cocktail (Complete Mini, Roche Diagnostics, Indianapolis, USA) and 0.04 U/mL aprotinin. After homogenisation and centrifugation at 1000 g for 5 min at 4°C, 20 µg of proteins (BCA assay) were separated on 12% SDS-PAGE gel electrophoresis, then transferred onto polyvinyl difluoride membranes (GE Healthcare Life Sciences, Uppsala, Sweden). The membranes were incubated with anti-AT_1_ receptor (1∶1000, 1 h, N10, Santa Cruz Biotechnology, Santa Cruz, USA), anti-AT_2_ receptor (1∶200, 2 h, H143, Santa Cruz) or anti-β-actin (1∶5000, 1 h, Sigma) polyclonal antibodies, followed by incubation (1 h) with the anti-rabbit IgG horseradish peroxidase antibody (1∶5000, Santa Cruz) at room temperature. Immunoreactive proteins were visualized on a Chemidoc apparatus (Biorad, Hercules, CA, USA) by chemiluminescence (peroxidase enzymatic reaction, ECL Western Blot detection system, GE Healthcare Life Sciences).

The amount of proteins was quantified (Quantity One, BioRad) and reported relatively to β-actin (n = 5 rats per group, duplicate). Measurement of AT_1_ and AT_2_ receptors protein levels has been validated in a previous study [16]. Representative Western-blots are provided on supplemental data online (Figure S1).

### Measurement of PPAR-gamma, eNOS and AT_1_ and AT_2_ receptors mRNA levels

Expressions of PPAR-gamma, eNOS and AT_1_ and AT_2_ receptors mRNA (n = 5 rats per group, duplicate) were measured following total RNA isolation of cerebral microvessels using Trizol Reagent (Sigma Aldrich). Quality of RNA extraction was determined by spectrophotometry (BioSpec-Nano, Shimadzu, Columbia, MD, USA) and RNA Nanochip (Agilent 2100 Bioanalyzer, Santa Clara, CA, USA). Reverse transcription (RT) was performed by standard methods (RNA to cDNA kit, Invitrogen, Cergy Pontoise, France). The quantitative polymerase chain reaction with synthetic gene-specific primers for PPAR-gamma, eNOS, AT_1_ and AT_2_ (sequences available on supplemental data online, Table S2, primers for AT_1_ and AT_2_ have been previously validated [16]), was performed using MESA GREEN qPCR MasterMix Plus for SYBR® Assay Low ROX (Eurogentec, Angers, France). The amplification was performed at 95°C for 10 min, followed by 50 cycles at 95°C for 15 s and 60°C for 30 s (Stratagene Mx3005P, Agilent Technologies, Santa Clara, CA, USA). To obtain a calibration curve, serial dilutions of rat kidney cDNA were used. The individual targets for each sample were quantified by determining the cycle threshold (Ct) and by using a calibration curve. The optimal set of housekeeping genes were selected from a series of candidate reference genes after geNorm® analysis. Two housekeeping genes selected, YWHAZ and HPRT1, were used for the determination of a normalization factor [27]. The level of expression of each gene was thus normalized and expressed relatively to WKY (study 1) or to SHR (study 2).

### Measurement of plasma ARB concentrations

At the end of the treatment periods and before sacrifying the animals, plasma was collected from arterial blood on EDTA tube and frozen at −80°C until analysis. TELMI and candesartan (active form of CANDE) were determined in rat plasma using an isocratic reversed-phase HPLC technique coupled with spectrofluorimetric detection. After protein precipitation with 1% acetonitrile (v/v in 1 M HCl), the resulting supernatant (20 µL) was loaded on the Spherisorb ODS (125×4 mm I.D.; 5-µm particle size) column and eluted at a flow rate of 1 mL/min and at a temperature of 40°C with (i) acetonitrile – 100 mmol/L phosphate buffer pH 6.0 (30∶70, v/v) for TELMI, (ii) acetonitrile – 0.74 mmol/L triethylammonium phosphate buffer pH 2.9 (30∶70, v/v) for candesartan. Detection was operated at λexc = 305 nm and λem = 365 nm for TELMI, and at λexc = 259 nm and λem = 392 nm for candesartan. Retention times were 5.2 and 8.4 min for TELMI and candesartan, respectively. Full selectivity *versus* endogenous compounds was observed. Recoveries were 96±9% for TELMI and 84±4% for candesartan. Linearity was tested in the range of 10 to 200 ng/mL for TELMI and 50 to 1000 ng/mL for candesartan. Intra-day relative standard deviation was 5.9% at a concentration of 50 ng/mL of TELMI (n = 5) and 5.3% at 500 ng/mL of candesartan (n = 6).

### Substances used

TELMI was provided by Boehringer Ingelheim Pharma GmbH & Co. KG (Ingelheim am Rhein, Germany), candesartan (standard for HPLC) and CANDE by the Astra Zeneca Company (Mölndal, Sweden) and PIO by Takeda (Chemicals Industries Ltd, Osaka, Japan). Sodium pentobarbitone was purchased from Sanofi-Aventis (Libourne, France) and all other reagents from Sigma Chemical Company (St Louis, MO, USA). For application in the cranial window, all drugs were dissolved in artificial cerebrospinal fluid [16], [20], [21], [23].

### Statistical analysis

Statistical analyses were performed using GraphPad Prism version 5 for Windows, (GraphPad Software, La Jolla California USA, www.graphpad.com). Results are expressed as means ± s.e.m.

#### Study 1

Four groups of rats were compared: normotensive rats WKY, vehicle-treated hypertensive rats SHR, SHR treated with CANDE and SHR treated with TELMI (TELMI). Significant differences between the groups were determined by a one-way ANOVA followed by a post-hoc Newman-Keuls test.

#### Study 2

Our groups of SHR were compared: vehicle-treated SHR, SHR treated with CANDE, PIO or a combination of both (CANDE+PIO). TELMI (TELMI) served as a positive control for AT_1_ receptor blockade combined with PPAR-gamma activity. Significant differences between SHR, CANDE, PIO and CANDE+PIO were determined by a two-way ANOVA (variables: “cande” and “pio”) followed by a post-hoc Bonferroni test. An interaction between cande and pio was considered significant if p value of interaction (p_interaction_) was <0.05. TELMI was compared to SHR, CANDE or CANDE+PIO using a two-tailed Student t-test. The null hypothesis was rejected at p<0.05.

## Results

### Study 1

**Figure 1:**
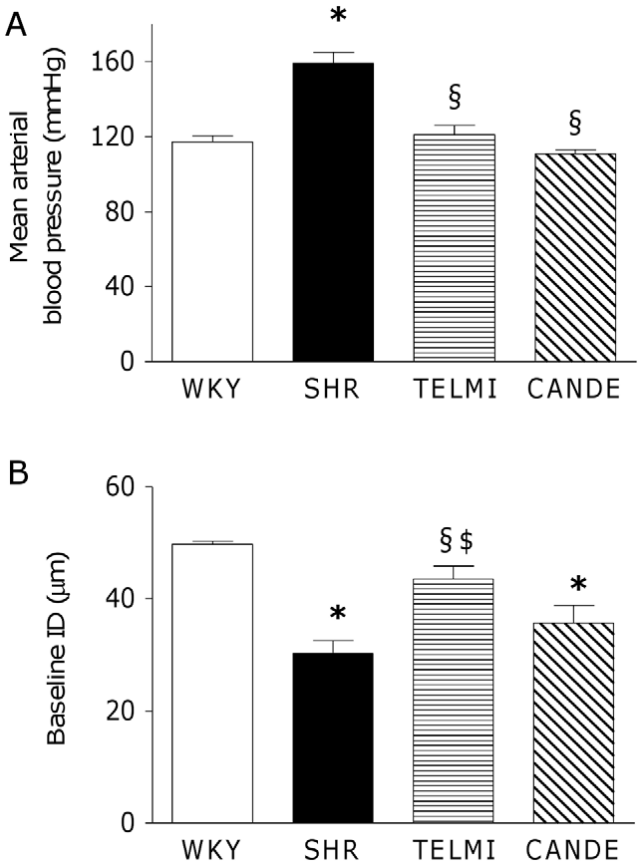
Blood pressure and basal pial arteriolar diameters. **A:** Systemic mean arterial blood pressure (mmHg) and **B**: baseline internal diameter of pial arterioles (ID, µm) in 4–5 month-old normotensive rats (WKY, empty bars), hypertensive rats that where vehicle-treated (SHR, full bars) or treated for 10 days with telmisartan (TELMI, 2 mg/kg per day, horizontal hatched bars) or candesartan cilexetil (CANDE, 10 mg/kg per day, left-sloping hatched bars). n = 5–7 per group, m±sem; p value for one-way ANOVA <0.0001; Newman-Keuls, *: p<0.05 *vs* WKY, § p<0.05 *vs* SHR, $ p<0.05 *vs* CANDE.

Systemic MAP was 36% higher in SHR. TELMI and CANDE restored MAP to the level of normotensive WKY rats (Figure 1A). Baseline ID was 39% lower in SHR than in WKY. TELMI (+44%), but not CANDE, increased baseline ID *versus* vehicle-treated SHR (Figure 1B).

**Figure 2:**
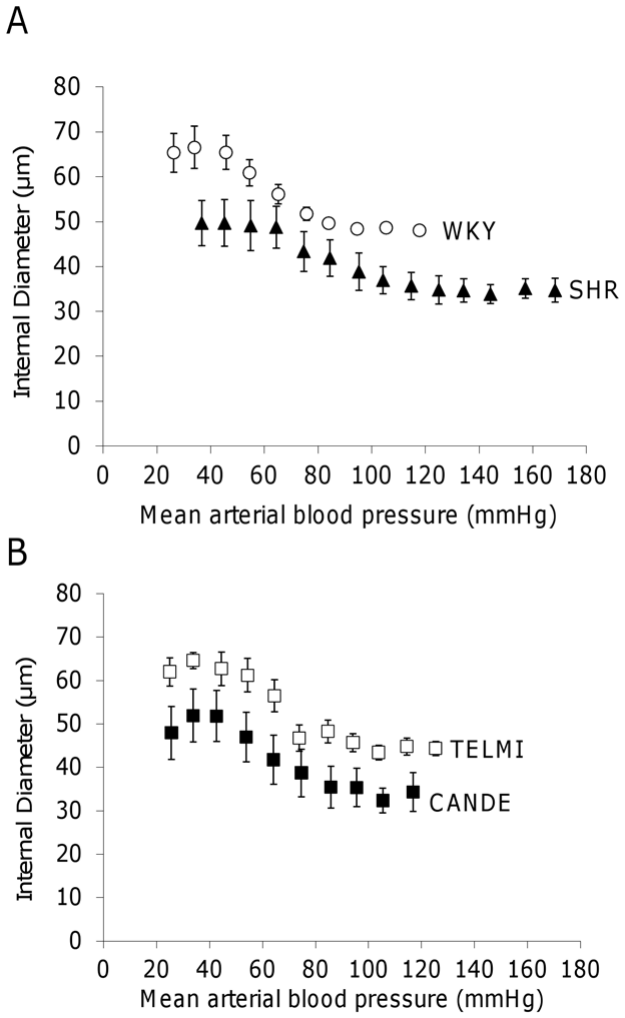
Hypotension-induced changes in pial arteriolar diameters. Internal diameters of pial arterioles (ID, µm) during decrease in systemic mean arterial blood pressure (mmHg), **A**: in 4–5 month-old normotensive rats (WKY, empty circles) and hypertensive vehicle-treated rats (SHR, full triangles); and **B**: in hypertensive rats treated for 10 days with telmisartan (TELMI, 2 mg/kg per day, empty squares) or candesartan cilexetil (CANDE, 10 mg/kg per day, full squares). n = 4–8 per group, m±sem.

For all steps of the hemorrhage-induced hypotension, ID of SHR and CANDE remained below those of WKY and TELMI (Figure 2).

**Figure 3:**
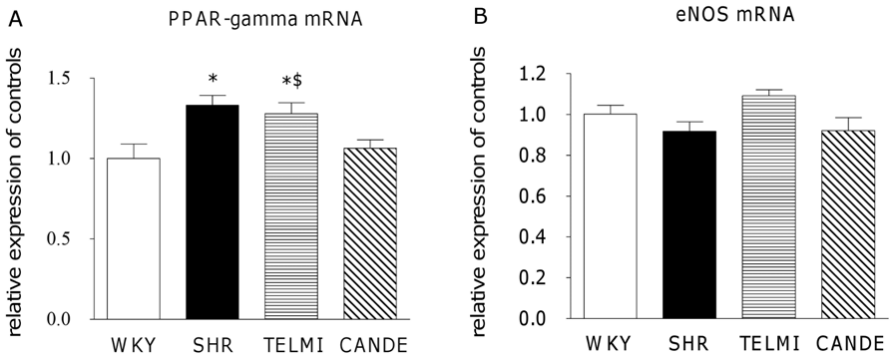
Levels of PPAR-gamma and eNOS mRNA expression. **A:** PPAR-gamma and **B:** eNOS mRNA expression in brain microvessels of 4–5 month-old normotensive rats (WKY, empty bars), hypertensive rats that where vehicle-treated (SHR, full bars) or treated for 10 days with telmisartan (TELMI, 2 mg/kg per day, horizontal hatched bars) or candesartan cilexetil (CANDE, 10 mg/kg per day, left-sloping hatched bars). n = 4–5, m±sem; 1-way ANOVA; Newman-Keuls *: p<0.05 *vs* WKY, $: p<0.05 *vs* CANDE.

PPAR-gamma mRNA expression was 33% higher in SHR than in WKY, remained higher in TELMI than in CANDE (Figure 3A). eNOS mRNA expression was not significantly different between groups (Figure 3B).

### Study 2

**Figure 4:**
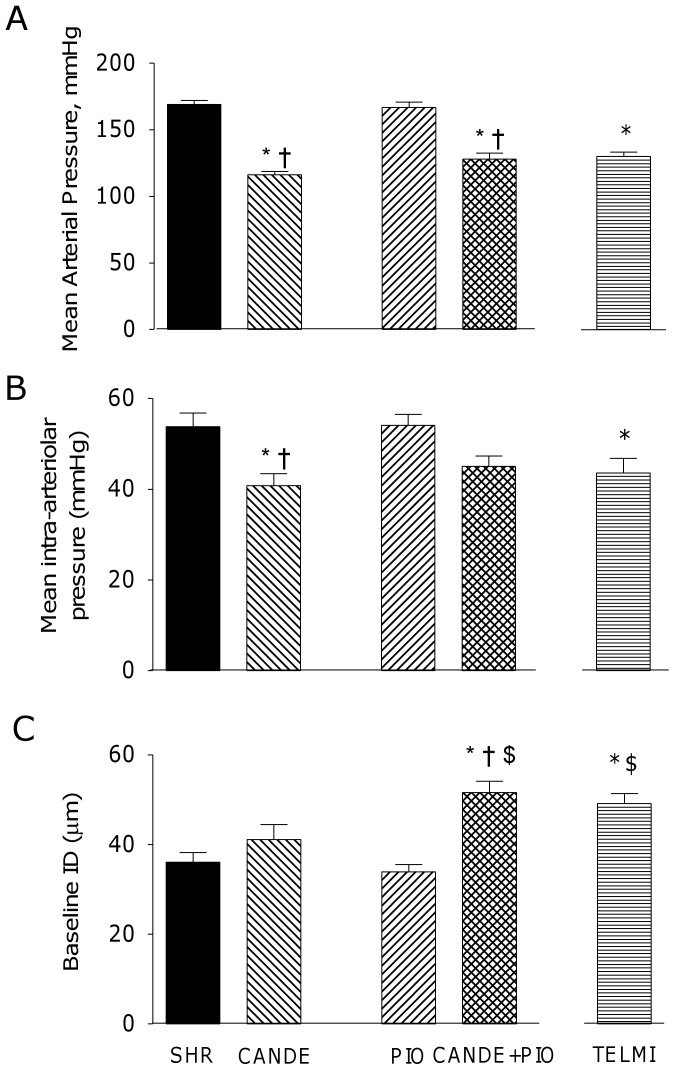
Blood pressure and baseline pial arteriolar diameters. **A:** Systemic mean arterial blood pressure (mmHg), **B:** mean intra-arteriolar blood pressure (mmHg) and **C:** baseline internal diameter of pial arterioles (ID, µm) in 4–5 month-old SHR that where vehicle-treated (SHR, full bars) or treated for 10 days with candesartan cilexetil (CANDE, 10 mg/kg per day, left-sloping hatched bars), pioglitazone (PIO, 2.5 mg/kg per day, right-sloping hatched bars) or both (CANDE+PIO, 10+2.5 mg/kg per day, double-sloping hatched bars), or telmisartan (TELMI, 2 mg/kg per day, horizontal hatched bars); m±sem. p values for two-way ANOVA: - A: mean arterial blood pressure (n = 13–18) p_interaction_ 0.051, p_cande_<1.10^−4^, p_pio_ 0.180 - B: mean intra-arteriolar blood pressure (n = 10–12) p_interaction_ 0.456, p_cande_<1.10^−4^, p_pio_ 0.389 – C: ID (n = 12–13) p_interaction_ 0.017, p_cande_<1.10^−4^, p_pio_ 0.107 Bonferroni post-test: * p<0.05 *vs* SHR, † p<0.05 *vs* PIO, $: p<0.05 *vs* CANDE t-tests for TELMI: * p<0.05 *vs* SHR, $: p<0.05 *vs* CANDE.

At the end of the treatment period, plasma concentration of candesartan was 170±15 ng/mL in CANDE and 175±18 ng/mL in CANDE+PIO, and that of TELMI was 54±2 ng/mL in TELMI. CANDE alone or in combination decreased systemic MAP and PiAP *versus* SHR, as did TELMI (Figure 4A, B). PIO had no antihypertensive effect.

CANDE+PIO (+43%) but neither CANDE nor PIO alone increased baseline ID of SHR (Figure 4C). Baseline ID in TELMI was greater than SHR (+36%) and CANDE (+20%) but not different from that of CANDE+PIO.

At 30–35 mmHg of intra-arteriolar pressure, CANDE+PIO but neither CANDE nor PIO alone, increased passive ID *versus* SHR (Table 1). CANDE, PIO and CANDE+PIO did not changed WT. Slope of the tangential elastic modulus *versus* stress was lower in CANDE+PIO than in CANDE (Table 1). Passive ID, WT and slope of the tangential elastic modulus *versus* stress in TELMI were not different from those of CANDE+PIO, CANDE or SHR (Table 1).

**Table 1:** Structure and vasoactivity of pial arterioles.

	SHR	CANDE	PIO	CANDE+PIO	2 way Anova			TELMI
					P_interaction_	P_cande_	P_pio_	
**Structure**								
Passive ID_30–35_, µm	77±5	85±4	73±5	100±6* †	0.087	0.004	0.345	89±4
WT_30–35_, µm	2.8±0.3	2.6±0.1	2.4±0.3	2.2±0.3	0.88	0.65	0.27	2.1±0.2
E_T_	5.3±0.5	7.4±0.8	6.0±0.5	4.6±0.4$	0.008	0.56	0.11	5.8±0.7
**Vasoactivity**								
5-HT 10^−6^ M,%	−10±1	−11±2	−11±1	−10±2	0.76	0.96	0.95	−10±1
5-HT 10^−6^+ADP 10^−4^ M,%	17±1	22±1	16±3	20±2	0.64	0.06	0.38	17±2

Passive internal diameter (passive ID, µm) and wall thickness at the arteriolar pressure range of 30–35 mmHg (WT_30–35_, µm) and slope of the elastic modulus *versus* stress (E_T_), responses of pial arterioles to serotonin and ADP (percentage of change in baseline ID) of 4–5 month-old SHR that where vehicle-treated (SHR) or treated for 10 days with candesartan cilexetil (CANDE, 10 mg/kg per day), pioglitazone (PIO, 2.5 mg/kg per day) or both (CANDE+PIO, 10+2.5 mg/kg per day), or telmisartan (TELMI, 2 mg/kg per day).

n = 6–15, m±sem, Bonferroni post-test:

* p<0.05 *vs* SHR,

† p<0.05 *vs* PIO,

$ : p<0.05 *vs* CANDE.

t-tests for TELMI: ! p<0.05 vs SHR, £: p < 0.05 vs CANDE, ¥: p < 0.05 vs CANDE+PIO.

Vasoconstrictor responses to 5-HT were similar in all groups, as were vasodilation responses to ADP in the presence of 5-HT (Table 1).

PPAR-gamma mRNA expression was not significantly different in SHR, CANDE, PIO and CANDE+PIO. PPAR-gamma expression in TELMI was higher than in CANDE (Table 2). Expression of eNOS mRNA remained stable in all groups, but not in TELMI (+20%). Treatments did not change reduced glutathione contents in brain microvessels (Table 2).

**Table 2:** Levels of PPAR-gamma and eNOS expression and glutathione content in brain microvessels.

	SHR	CANDE	PIO	CANDE +PIO	2 way Anova			TELMI
					P_interaction_	P_cande_	P_pio_	
PPAR-gamma mRNA	1.00±0.04	0.80±0.03	1.0±0.1	0.9±0.1	0.57	0.22	0.35	0.96±0.04£
eNOS mRNA	1.00±0.05	0.99±0.07	1.00±0.03	1.00±0.05	0.97	0.94	0.91	1.18±0.03! £ ¥
GSH, nmol/mg protein	2.5±0.4	2.3±0.1	2.7±0.3	3.2±0.4	0.39	0.73	0.20	2.6±2.3

PPAR-gamma and eNOS mRNA expression in brain microvessels and glutathione (GSH) content of brain microvessels of 4–5 month-old SHR that where vehicle-treated (SHR) or treated for 10 days with candesartan cilexetil (CANDE, 10 mg/kg per day), pioglitazone (PIO, 2.5 mg/kg per day) or both (CANDE+PIO, 10+2.5 mg/kg per day), or telmisartan (TELMI, 2 mg/kg per day).

n = 6–15, m±sem, Bonferroni post-test: $: p<0.05 *vs* CANDE.

t-tests for TELMI:

! p<0.05 *vs* SHR,

£ : p<0.05 *vs* CANDE,

¥ : p<0.05 *vs* CANDE+PIO.

**Figure 5:**
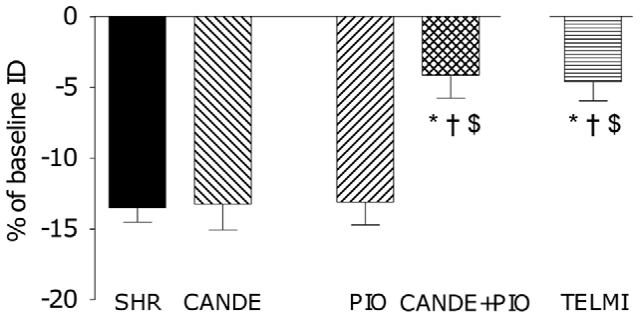
Vasoreactivity of pial arterioles to Ang II. Vasoactive response of pial arterioles (percentage of change in baseline ID) to suffusion of Ang II (10^−6^ M) in 4–5 month-old SHR that where vehicle-treated (SHR, full bars) or treated for 10 days with candesartan cilexetil (CANDE, 10 mg/kg per day, left-sloping hatched bars), pioglitazone (PIO, 2.5 mg/kg per day, right-sloping hatched bars) or both (CANDE+PIO, 10+2.5 mg/kg per day, double-sloping hatched bars), or telmisartan (TELMI, 2 mg/kg per day, horizontal hatched bars) ; m±sem. p values for two-way ANOVA (n = 7–9) p_interaction_ 0.018, p_cande_ 0.012, p_pio_ 0.009 Bonferroni post-test: * p<0.05 *vs* SHR, † p<0.05 *vs* PIO, $: p<0.05 *vs* CANDE t-tests for TELMI: * p<0.05 *vs* SHR, † p<0.05 *vs* PIO, $: p<0.05 *vs* CANDE.

Vasoconstrictor responses to Ang II were similar in SHR, PIO and CANDE. Only CANDE+PIO decreased this response when compared to SHR (Figure 5, p_interaction_ = 0.018). TELMI was as effective as CANDE+PIO to reduce Ang II-induced vasoconstriction. None of the treatments had any significant impact on AT_1_ and AT_2_ receptors mRNA and protein expressions in brain microvessels (see data on the online supplementary file, Figure S2).

## Discussion

For similar antihypertensive effects, short-term treatment with TELMI or CANDE in combination with PIO, but not CANDE alone, reverses hypertension-induced narrowing of pial arteriolar ID. This may involve a synergistic action between AT_1_ receptor blockade and PPAR-gamma actions, and also a better AT_1_ receptor blockade.

### Functional remodeling

Ten-day treatment with TELMI, but not with CANDE, restored baseline ID to the level of normotensive rats. This suggests an early reversal effect of the chronic hypertension-induced remodeling of pial arterioles by TELMI. During hemorrhage, ID in TELMI and WKY reached similar values, while those of SHR and CANDE remained lower. Compared to CANDE, the effect of TELMI could constitute a major protective mechanism for maintenance of cerebral blood flow during hypotensive situations such as ischemic stroke. In another study in SHR, CANDE progressively normalized cerebral autoregulation with a better cerebral blood flow than vehicle-treated SHR, but this appeared only after 14 days of treatments [7]. In the present studies, functional remodeling did not contribute to changes in ID, as responses to vasoactive agents were not changed (except that to Ang II) by any of the treatments.

### Structural remodeling and role of blood pressure reduction

For similar antihypertensive effect (restoration to WKY level), similar reversal of hypertension-induced structural remodeling would be expected in all groups treated with ARBs. This was not the case in the present study, as only CANDE+PIO changed passive ID *versus* SHR.

First, the ten-day reduction in arteriolar pressure is probably too short to allow significant structural changes. Moreover, blood pressure reduction may not be sufficient by itself to increase ID. For example an antihypertensive dose of beta-blocker did not prevent pial arteriolar remodeling [28] and, in the present study, CANDE decreased arteriolar pressure but failed to increase ID. The latter is associated with a lack of blockade of the angiotensin II response at the pial arteriolar level. Several reports show that chronic blockade of the renin angiotensin system is a major determinant of the reversal of cerebrovascular remodeling. Increases in ID have been observed with non-antihypertensive low doses of perindopril [28] or TELMI [29]. Our previous report with low dose of TELMI [21] showed no remodeling effect. However, this low dose did not block Ang II response at the pial arteriolar level. Altogether, these results suggest that AT_1_ receptor blockade is required to prevent hypertension-induced remodeling. For short-term treatments, this seems further amplified by PPAR-gamma agonist activity as CANDE+PIO significantly increased passive ID.

Besides, the pial arteriolar wall in CANDE+PIO was more distensible than in CANDE alone. This was mainly due to the increase in E_T_ in CANDE as compared to the other groups, rather than to a beneficial effect of CANDE+PIO. Even if the impact on baseline ID is questionable, such sustained distensibility in CANDE+PIO could afford a better adaptive capacity under major dilatory stimuli.

### Role of PPAR-gamma agonistic action

Our first hypothesis to explain the differential effects between short-term treatment with TELMI and CANDE relies on the level of PPAR-gamma activation produced by TELMI [8]. To demonstrate this hypothesis, we initially supplemented TELMI with bisphenol A diglycidyl ether, a PPAR-gamma antagonist (BADGE, Sigma D3415, [30]). However, we had to stop as BADGE slowly decreased arterial pressure (data not shown), making results difficult to interpret. We thus used the other strategy to combine CANDE, described to have no PPAR-gamma properties [8], and PIO. This combination revealed a synergistic action on baseline ID.

Brain microvessels from SHR showed an increased PPAR-gamma expression compared to WKY in study 1. This has been reported in blood vessels as a compensatory mechanism. Indeed, pleiotropic actions of PPAR-gamma reduce cell growth, inflammation, oxidative stress and endothelial dysfunction related to hypertension [31]–[33]. This protective expression of PPAR-gamma was maintained in TELMI but not in CANDE, whereas both treatments suppressed hypertension. In parallel, TELMI, but not CANDE, increased eNOS expression, a target gene for PPAR-gamma activity in vascular tissues [10], [11]. However, the increase of eNOS expression was not associated with any improvement of endothelial function (evaluated *via* ADP reactivity) in TELMI. This may be due to the lack of endothelial dysfunction in SHR in the present study (ADP responses: SHR 17±1 *versus* WKY 17±3%, p>0.05) as previously reported [34]. In order to clarify the implication of PPAR-gamma activity, CANDE was combined with PIO. We observed the same beneficial effect on pial arteriolar ID as in TELMI, but with some discrepancies. First, PIO did not modify PPAR-gamma nor eNOS expressions in PIO and CANDE+PIO. Such an absence of modification in eNOS expression has been already observed following a longer treatment with another PPAR-gamma agonist [35]. Furthermore, we did not observed any impact of PIO on the remodeling of pial arterioles in SHR. This is in contrast with previously observed beneficial effects on structure and extracellular matrix composition in larger arteries [12], [36]. This could be related to (i) the low severity of the present model (SHR *versus* SHR-SP [34] or a rat model of strong arteriosclerosis induced by elastocalcinosis [12]) and (ii) the short duration of treatment (10 days treatment *versus* 6 or 4 weeks [12], [36]). Finally, in the present study, oxidative stress (or its reversion) did not contribute to the hypertension- or treatment-induced changes in ID as we did not find any changes in reduced glutathione content inside of brain microvessels.

### Role of AT_1_ receptor blockade

A second hypothesis to explain the effect of TELMI or CANDE+PIO on baseline ID may rely on a differential blockade of AT_1_ receptors. Inhibition of the vasoconstrictor response to Ang II in TELMI and CANDE+PIO, but not in CANDE alone, despite similar plasma concentrations of candesartan in CANDE and CANDE+PIO, supports this hypothesis. As the mRNA and protein expressions of both AT_1_ and AT_2_ receptors did not change, this cannot be responsible for the differences in vasoreactivity to Ang II. The other possibility may be a lower penetration of the blood brain barrier and a lower concentration of CANDE reaching the arteriolar smooth muscle cells compared to TELMI. CANDE is known to induce cerebral effects [37], but it has not been detected in cerebral tissues when administered at a similar dose and duration as in this study [38]. The lower lipophilic properties of CANDE as compared to TELMI may play a role in blood brain barrier crossing ability (logP_cande_ = 4.6±0.7 *versus* logP_telmi_ = 7.1±0.9; evaluated by ALOGPS 2.1 program, Virtual Computational Chemistry Laboratory http://www.vcclab.org). As the PPAR-gamma agonistic activity is implied in blood brain barrier permeability [39], [40], this might be involved (through an unknown mechanism) in the improvement of crossing properties of the drugs in CANDE+PIO, and explain why CANDE+PIO is more effective than CANDE to inhibit Ang II. This, however, remains to be tested on a blood brain barrier model. The PPAR-gamma agonist activity of TELMI could also explain why TELMI is as effective as CANDE+PIO.

In summary, we initiated a short-term treatment in young adult SHR in which hypertension already induced structural remodeling without strong vasoactive dysfunction. Despite the low severity of the model and a short-term period of treatment, combination of CANDE and PIO allowed reversal of pial arteriolar remodeling, as TELMI. This synergistic action between AT_1_ receptor blockade and PPAR-gamma actions may result from the important degree of crosstalk between the renin angiotensin and PPAR-gamma systems [41]–[43]. Therefore, ten-day treatment with TELMI or a combination of CANDE and PIO, but not CANDE alone, reverses the hypertension-induced narrowing of pial arterioles despite a similar reduction in blood pressure. This could be related to both (i) PPAR-gamma activation and (ii) differences in the degree of AT_1_ receptor blockade at the pial arteriolar level. Our results suggest that addition of PPAR-gamma activation to AT_1_ blockade could exert or accelerate the effect of ARBs on hypertension-induced narrowing of pial arteriolar diameter. This differential effect of short-term treatment according to ARBs could be of particular interest in post-stroke situations where a rapid effect is required to improve the outcome of patients.

## Supporting Information

Figure S1 **Representative Western-blots.** Representative Western-blots of proteins from brain microvessels with A: anti-actin antibody; B: anti-AT_1_ receptor antibody; C: anti-AT_2_ receptor antibody. W: WKY; S: SHR; T: TELMI; C: CANDE; CP: CANDE+PIO; +^1^: lysates from NIH/3T3 cells in which AT_1_ receptors are highly expressed (positive control for AT_1_ receptor expression); +^2^: lysates from KNRK cells in which AT_2_ receptors are highly expressed (positive control for AT_2_ receptor expression). Image acquisition of immunoreactive proteins was performed on a Chemidoc apparatus (Biorad, Hercules, CA, USA).
(TIFF)

Figure S2 **Levels of AT_1_ and AT_2_ receptors expression in brain microvessels.** mRNA and protein expressions of AT_1_ and AT_2_ receptors in cerebral microvessels isolated from 4–5 month-old SHR that where untreated (SHR, full bars) or treated for 10 days with candesartan cilexetil (CANDE, 10 mg/kg perday, left-sloping hatched bars), pioglitazone (PIO, 2.5 mg/kg per day, right-sloping hatched bars) or both (CANDE+PIO, 10+2.5 mg/kg per day, double-sloping hatched bars), or telmisartan (TELMI, 2 mg/kg per day, horizontal hatched bars); m±sem. p values for two-way ANOVA: - AT_1_ receptor mRNA (n = 4–5) p_interaction_ 0.22, p_cande_ 0.26, p_pio_ 0.09 - AT_2_ receptor mRNA (n = 4–5) p_interaction_ 0.40, p_cande_ 0.73, p_pio_ 0.09 - AT_1_ receptor protein (n = 4) p_interaction_ 0.94, p_cande_ 0.09, p_pio_0.86 - AT_2_ receptor protein (n = 4) p_interaction_ 0.89, p_cande_ 0.69, p_pio_ 0.46.
(TIFF)

Table S1 **Initial values of pH and arterial blood gases (m±sem).**
(DOCX)

Table S2 **Primers sequences for quantitative polymerase chain reaction**
(DOCX)

## References

[1] Diener HC (2009) Preventing stroke: the PRoFESS, ONTARGET, and TRANSCEND trial programs. J Hypertens Suppl 27: S31–S36. 19587553 10.1097/01.hjh.0000357906.60778.7f

[2] Trenkwalder P (2006) The Study on COgnition and Prognosis in the Elderly (SCOPE)-recent analyses. J Hypertens Suppl 24: S107–S114. 16601563 10.1097/01.hjh.0000220415.99610.22

[3] Ando H, Zhou J, Macova M, Imboden H, Saavedra JM (2004) Angiotensin II AT1 receptor blockade reverses pathological hypertrophy and inflammation in brain microvessels of spontaneously hypertensive rats. Stroke 35: 1726–1731. 15143297 10.1161/01.STR.0000129788.26346.18

[4] Dupuis F, Atkinson J, Limiñana P, Chillon JM (2005) Comparative effects of the angiotensin II receptor blocker, telmisartan, and the angiotensin-converting enzyme inhibitor, ramipril, on cerebrovascular structure in spontaneously hypertensive rats. J Hypertens 23: 1061–1066. 15834293 10.1097/01.hjh.0000166848.95592.a5

[5] Zhou J, Ando H, Macova M, Dou J, Saavedra JM (2005) Angiotensin II AT1 receptor blockade abolishes brain microvascular inflammation and heat shock protein responses in hypertensive rats. J Cereb Blood Flow Metab 25: 878–888. 15729290 10.1038/sj.jcbfm.9600082

[6] Sandset EC, Bath PMW, Boysen G, Jatuzis D, Kõrv J, et al (2011) The angiotensin-receptor blocker candesartan for treatment of acute stroke (SCAST): a randomised, placebo-controlled, double-blind trial. Lancet 377: 741–750. 21316752 10.1016/S0140-6736(11)60104-9

[7] Nishimura Y, Ito T, Saavedra JM, Angiotensin AT II (1) blockade normalizes cerebrovascular autoregulation and reduces cerebral ischemia in spontaneously hypertensive rats (2000). Stroke 31: 2478–2486. 11022082 10.1161/01.str.31.10.2478

[8] Benson SC, Pershadsingh HA, Ho CI, Chittiboyina A, Desai P, et al (2004) Identification of telmisartan as a unique angiotensin II receptor antagonist with selective PPARgamma-modulating activity. Hypertension 43: 993–1002. 15007034 10.1161/01.HYP.0000123072.34629.57

[9] Schupp M, Janke J, Clasen R, Unger T, Kintscher U (2004) Angiotensin type 1 receptor blockers induce peroxisome proliferator-activated receptor-gamma activity. Circulation 109: 2054–2057. 15117841 10.1161/01.CIR.0000127955.36250.65

[10] Yuen CY, Wong WT, Tian XY, Wong SL, Lau CW, et al (2011) Telmisartan inhibits vasoconstriction via PPARγ-dependent expression and activation of endothelial nitric oxide synthase. Cardiovasc Res 90: 122–129. 21156825 10.1093/cvr/cvq392

[11] Kobayashi N, Ohno T, Yoshida K, Fukushima H, Mamada Y, et al (2008) Cardioprotective mechanism of telmisartan via PPAR-gamma-eNOS pathway in dahl salt-sensitive hypertensive rats. Am J Hypertens 21: 576–581. 18437150 10.1038/ajh.2008.27

[12] Gaillard V, Casellas D, Seguin-Devaux C, Schohn H, Dauca M, et al (2005) Pioglitazone improves aortic wall elasticity in a rat model of elastocalcinotic arteriosclerosis. Hypertension 46: 372–379. 15967870 10.1161/01.HYP.0000171472.24422.33

[13] Diep QN, El Mabrouk M, Cohn JS, Endemann D, Amiri F, et al (2002) Structure, endothelial function, cell growth, and inflammation in blood vessels of angiotensin II-infused rats: role of peroxisome proliferator-activated receptor-gamma. Circulation 105: 2296–2302. 12010913 10.1161/01.cir.0000016049.86468.23

[14] Beyer AM, Baumbach GL, Halabi CM, Modrick ML, Lynch CM, et al (2008) Interference with PPARgamma signaling causes cerebral vascular dysfunction, hypertrophy, and remodeling. Hypertension 51: 867–871. 18285614 10.1161/HYPERTENSIONAHA.107.103648 PMC2408877

[15] Iwanami J, Mogi M, Tsukuda K, Min L-J, Sakata A, et al (2010) Low dose of telmisartan prevents ischemic brain damage with peroxisome proliferator-activated receptor-gamma activation in diabetic mice. J Hypertens 28: 1730–1737. 20498620 10.1097/HJH.0b013e32833a551a

[16] Foulquier S, Dupuis F, Perrin-Sarrado C, Maguin Gate K, Merhi-Soussi F, et al (2011) High salt intake abolishes AT(2)-mediated vasodilation of pial arterioles in rats. J Hypertens 29: 1392–1399. 21519278 10.1097/HJH.0b013e328347050e

[17] Seltzer A, Bregonzio C, Armando I, Baiardi G, Saavedra JM (2004) Oral administration of an AT1 receptor antagonist prevents the central effects of angiotensin II in spontaneously hypertensive rats. Brain Res 1028: 9–18. 15518636 10.1016/j.brainres.2004.06.079

[18] Sugiyama Y, Taketomi S, Shimura Y, Ikeda H, Fujita T (1990) Effects of pioglitazone on glucose and lipid metabolism in Wistar fatty rats. Arzneimittelforschung 40: 263–267. 2189419

[19] Li YQ, Ji H, Zhang YH, Shi WB, Meng ZK, et al (2007) WB1106, a novel nitric oxide-releasing derivative of telmisartan, inhibits hypertension and improves glucose metabolism in rats. Eur J Pharmacol 577: 100–108. 17822696 10.1016/j.ejphar.2007.08.008

[20] Vincent JM, Kwan YW, Chan SL, Perrin-Sarrado C, Atkinson J, et al (2005) Constrictor and dilator effects of angiotensin II on cerebral arterioles. Stroke 36: 2691–2695. 16269635 10.1161/01.STR.0000190002.79052.bf

[21] Dupuis F, Vincent JM, Liminana P, Chillon JM, Capdeville-Atkinson C, et al (2010) Effects of suboptimal doses of the AT1 receptor blocker, telmisartan, with the angiotensin-converting enzyme inhibitor, ramipril, on cerebral arterioles in spontaneously hypertensive rat. J Hypertens 28: 1566–1573. 20589978 10.1097/hjh.0b013e328339f1f3

[22] Fog M (1937) Cerebral circulation. The reaction of the pial arteries to a fall of blood pressure. Arch Neurol Psychiatry 37: 351–364.

[23] Chan S-L, Tabellion A, Bagrel D, Perrin-Sarrado C, Capdeville-Atkinson C, et al (2008) Impact of chronic treatment with red wine polyphenols (RWP) on cerebral arterioles in the spontaneous hypertensive rat. J Cardiovasc Pharmacol 51: 304–310. 18356696 10.1097/FJC.0b013e318163a946

[24] Maguin Gate K, Lartaud I, Giummelly P, Legrand R, Pompella A, et al (2011) Accurate measurement of reduced glutathione in gamma-glutamyltransferase-rich brain microvessel fractions. Brain Res 1369: 95–102. 21047497 10.1016/j.brainres.2010.10.100

[25] Yamakawa H, Jezova M, Ando H, Saavedra JM (2003) Normalization of endothelial and inducible nitric oxide synthase expression in brain microvessels of spontaneously hypertensive rats by angiotensin II AT1 receptor inhibition. J. Cereb. Blood Flow Metab 23: 371–380. 10.1097/01.WCB.0000047369.05600.03 12621312

[26] Agarwal R, Shukla GS (1999) Potential role of cerebral glutathione in the maintenance of blood-brain barrier integrity in rat. Neurochem Res 24: 1507–1514. 10591399 10.1023/a:1021191729865

[27] Vandesompele J, De Preter K, Pattyn F, Poppe B, Van Roy N, et al (2002) Accurate normalization of real-time quantitative RT-PCR data by geometric averaging of multiple internal control genes. Genome Biol 3: research0034.0031–research0034.0011. 12184808 10.1186/gb-2002-3-7-research0034 PMC126239

[28] Chillon JM, Baumbach GL (1999) Effects of an angiotensin-converting enzyme inhibitor and a beta-blocker on cerebral arterioles in rats. Hypertension 33: 856–861. 10082499 10.1161/01.hyp.33.3.856

[29] Kumai Y, Ooboshi H, Ago T, Ishikawa E, Takada J, et al (2008) Protective effects of angiotensin II type 1 receptor blocker on cerebral circulation independent of blood pressure. Exp Neurol 210: 441–448. 18177860 10.1016/j.expneurol.2007.11.028

[30] Wright HM, Clish CB, Mikami T, Hauser S, Yanagi K, et al (2000) A synthetic antagonist for the peroxisome proliferator-activated receptor gamma inhibits adipocyte differentiation. J Biol Chem 275: 1873–1877. 10636887 10.1074/jbc.275.3.1873

[31] Diep QN, Schiffrin EL (2001) Increased expression of peroxisome proliferator-activated receptor-alpha and -gamma in blood vessels of spontaneously hypertensive rats. Hypertension 38: 249–254. 11509485 10.1161/01.hyp.38.2.249

[32] Touyz RM, Schiffrin EL (2006) Peroxisome proliferator-activated receptors in vascular biology-molecular mechanisms and clinical implications. Vascul Pharmacol 45: 19–28. 16782410 10.1016/j.vph.2005.11.014

[33] Sigmund CD (2010) Endothelial and vascular muscle PPARgamma in arterial pressure regulation: lessons from genetic interference and deficiency. Hypertension 55: 437–444. 20038751 10.1161/HYPERTENSIONAHA.109.144170 PMC2819308

[34] Kagota S, Tamashiro A, Yamaguchi Y, Sugiura R, Kuno T, et al (2001) Downregulation of vascular soluble guanylate cyclase induced by high salt intake in spontaneously hypertensive rats. Br J Pharmacol 134: 737–744. 11606313 10.1038/sj.bjp.0704300 PMC1572996

[35] Ryan MJ, Didion SP, Mathur S, Faraci FM, Sigmund CD (2004) PPAR(gamma) agonist rosiglitazone improves vascular function and lowers blood pressure in hypertensive transgenic mice. Hypertension 43: 661–666. 14744930 10.1161/01.HYP.0000116303.71408.c2

[36] Nakamura T, Yamamoto E, Kataoka K, Yamashita T, Tokutomi Y, et al (2007) Pioglitazone exerts protective effects against stroke in stroke-prone spontaneously hypertensive rats, independently of blood pressure. Stroke 38: 3016–3022. 17885259 10.1161/STROKEAHA.107.486522

[37] Gohlke P, Von Kugelgen S, Jurgensen T, Kox T, Rascher W, et al (2002) Effects of orally applied candesartan cilexetil on central responses to angiotensin II in conscious rats. J Hypertens 20: 909–918. 12011652 10.1097/00004872-200205000-00026

[38] Pelisch N, Hosomi N, Ueno M, Masugata H, Murao K, et al (2010) Systemic candesartan reduces brain angiotensin II via downregulation of brain renin-angiotensin system. Hypertens Res 33: 161–164. 19942928 10.1038/hr.2009.200 PMC2818705

[39] Roberts TJ, Chapman AC, Cipolla MJ (2009) PPAR-gamma agonist rosiglitazone reverses increased cerebral venous hydraulic conductivity during hypertension. Am J Physiol Heart Circ Physiol 297: H1347–H1353. 19666838 10.1152/ajpheart.00630.2009 PMC2770757

[40] Bian C, Wu Y, Chen P (2009) Telmisartan increases the permeability of endothelial cells through zonula occludens-1. Biol Pharm Bull 32: 416–420. 19252288 10.1248/bpb.32.416

[41] Zorad S, Dou J, Benicky J, Hutanu D, Tybitanclova K, et al (2006) Long-term angiotensin II AT1 receptor inhibition produces adipose tissue hypotrophy accompanied by increased expression of adiponectin and PPARgamma. EurJ Pharmacol 552: 112–122. 17064684 10.1016/j.ejphar.2006.08.062 PMC1764497

[42] Kuipers I, van der Harst P, Navis G, van Genne L, Morello F, et al (2008) Nuclear hormone receptors as regulators of the renin-angiotensin-aldosterone system. Hypertension 51: 1442–1448. 18413492 10.1161/HYPERTENSIONAHA.107.108530

[43] Xiao J, Leung JCK, Chan LYY, Tang SCW, Lai KN (2009) Crosstalk between peroxisome proliferator-activated receptor-gamma and angiotensin II in renal tubular epithelial cells in IgA nephropathy. Clin Immunol 132: 266–276. 19443277 10.1016/j.clim.2009.04.004

