# Supplementary figures and images for: Differential Effects of Short-Term Treatment with Two AT1 Receptor Blockers on Diameter of Pial Arterioles in SHR
Source: PLoS One. 2012 Sep 5;7(9):e42469. doi: 10.1371/journal.pone.0042469 (PMC3434186; doi:10.1371/journal.pone.0042469)

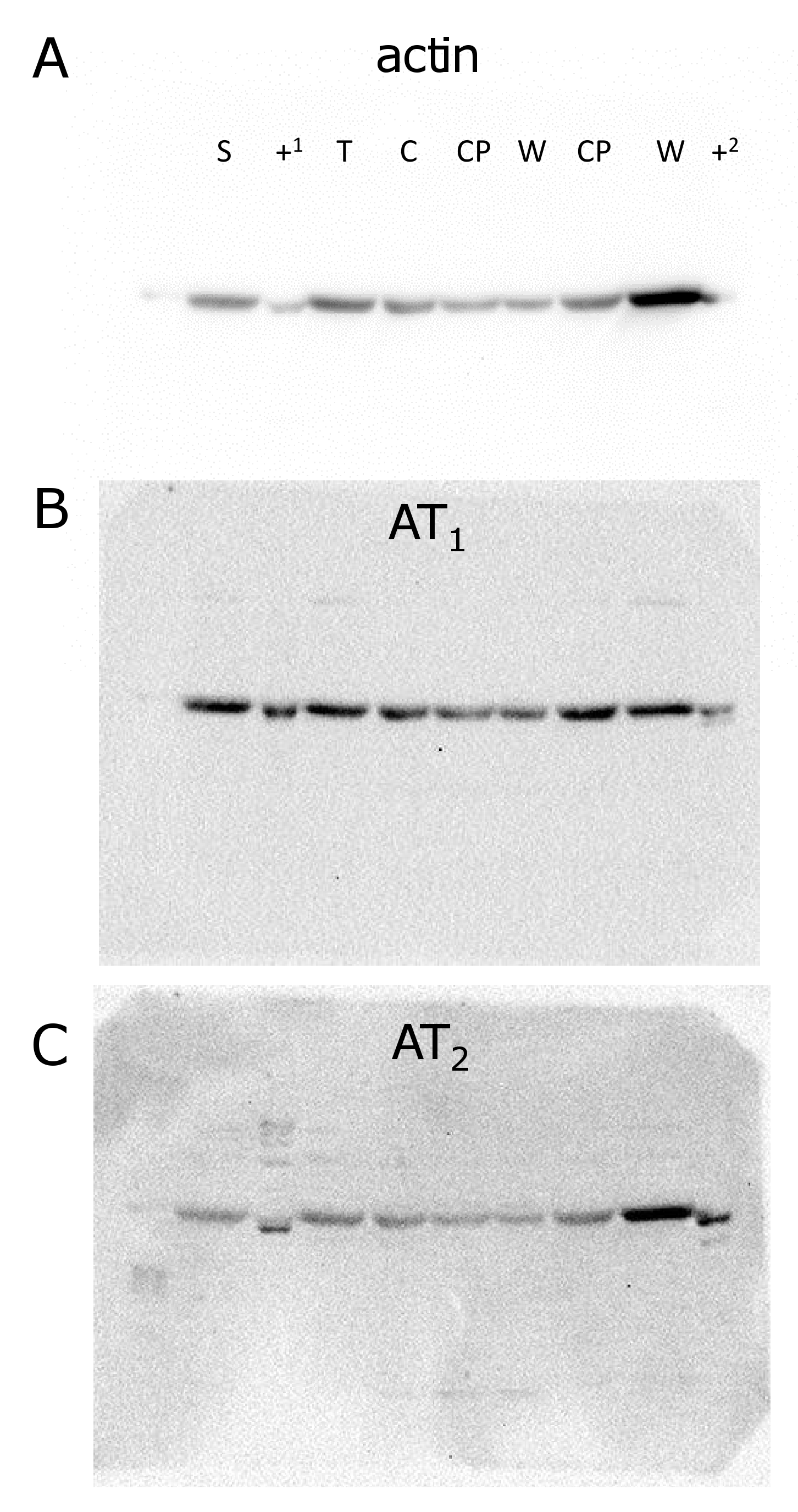

Supplement: Figure S1 — Representative Western-blots. Representative Western-blots of proteins from brain microvessels with A: anti-actin antibody; B: anti-AT1 receptor antibody; C: anti-AT2 receptor antibody. W: WKY; S: SHR; T: TELMI; C: CANDE; CP: CANDE+PIO; +1: lysates from NIH/3T3 cells in which AT1 receptors are highly expressed (positive control for AT1 receptor expression); +2: lysates from KNRK cells in which AT2 receptors are highly expressed (positive control for AT2 receptor expression). Image acquisition of immunoreactive proteins was performed on a Chemidoc apparatus (Biorad, Hercules, CA, USA). (TIFF) [file pone.0042469.s001.tiff]

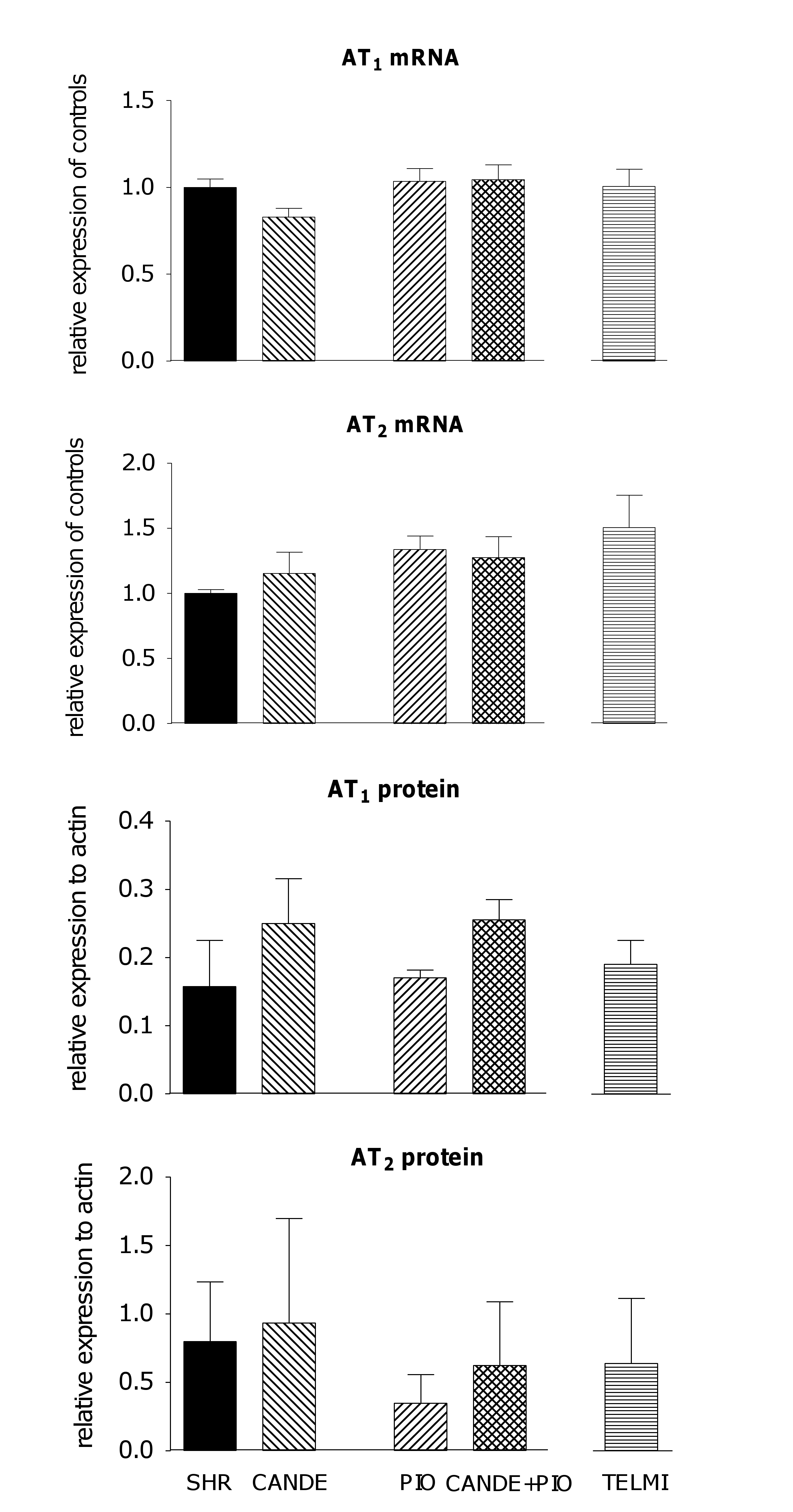

Supplement: Figure S2 — Levels of AT1 and AT2 receptors expression in brain microvessels. mRNA and protein expressions of AT1 and AT2 receptors in cerebral microvessels isolated from 4–5 month-old SHR that where untreated (SHR, full bars) or treated for 10 days with candesartan cilexetil (CANDE, 10 mg/kg perday, left-sloping hatched bars), pioglitazone (PIO, 2.5 mg/kg per day, right-sloping hatched bars) or both (CANDE+PIO, 10+2.5 mg/kg per day, double-sloping hatched bars), or telmisartan (TELMI, 2 mg/kg per day, horizontal hatched bars); m±sem. p values for two-way ANOVA: - AT1 receptor mRNA (n = 4–5) pinteraction 0.22, pcande 0.26, ppio 0.09 - AT2 receptor mRNA (n = 4–5) pinteraction 0.40, pcande 0.73, ppio 0.09 - AT1 receptor protein (n = 4) pinteraction 0.94, pcande 0.09, ppio0.86 - AT2 receptor protein (n = 4) pinteraction 0.89, pcande 0.69, ppio 0.46. (TIFF) [file pone.0042469.s002.tiff]
