# Supplementary material for: Differential Effects of Short-Term Treatment with Two AT1 Receptor Blockers on Diameter of Pial Arterioles in SHR
Source: PLoS One. 2012 Sep 5;7(9):e42469. doi: 10.1371/journal.pone.0042469 (PMC3434186; doi:10.1371/journal.pone.0042469)
Supplement: Table S1 — Initial values of pH and arterial blood gases (m±sem). (DOCX) [file pone.0042469.s003.docx]

Table S1: **Initial values of pH and arterial blood gases (m±sem)**

|  | **WKY** | **SHR** | **TELMI** | **CANDE** | **PIO** | **CANDE+PIO** |
| --- | --- | --- | --- | --- | --- | --- |
| **pH** | 7.34 ± 0.06 | 7.37 ± 0.03 | 7.41 ± 0.06 | 7.34 ± 0.03 | 7.28 ± 0.04 | 7.36 ± 0.06 |
| **pCO_2_, mmHg** | 35 ± 2 | 37 ± 1 | 36 ± 2 | 35 ± 1 | 38 ± 2 | 35 ± 2 |
| **pO_2_, mmHg** | 121 ± 7 | 140 ± 8 | 133 ± 7 | 141 ± 4 | 127 ± 5 | 129 ± 3 |
