# Supplementary material for: Differential Effects of Short-Term Treatment with Two AT1 Receptor Blockers on Diameter of Pial Arterioles in SHR
Source: PLoS One. 2012 Sep 5;7(9):e42469. doi: 10.1371/journal.pone.0042469 (PMC3434186; doi:10.1371/journal.pone.0042469)
Supplement: Table S2 — Primers sequences for quantitative polymerase chain reaction (DOCX) [file pone.0042469.s004.docx]

Table S2: **Primers sequences for quantitative polymerase chain reaction**

| ***Genes*** | ***Sense sequences*** | ***Antisense sequences*** |
| --- | --- | --- |
| ***PPAR-gamma*** | 5’-cccaatggttgctgattaca-3’ | 5’-ggacgcaggctctactttga-3’ |
| ***eNOS*** | 5’-tgaccctcaccgatacaaca-3’ | 5’-cgggtgtctagatccatgc-3’ |
| ***AT_1_*** | 5’-CACAGTGTGCGCGTTTCATT-3’ | 5’-TGGTAAGGCCCAGCCCTAT-3’ |
| ***AT_2_*** | 5’-Gaacagaattacccgtgacca-3’ | 5’-ATGAATGCCAACACAACAGC-3’ |
| ***YWHAZ*** | 5’-ctaccgctacttggctgagg-3’ | 5’- tgtgactggtccacaattcc -3’ |
| ***HPRT1*** | 5’-gaccggttctgtcatgtcg-3’ | 5’acctggttcatcatcactaatcac-3’ |
